# The use of AI in epilepsy and its applications for people with intellectual disabilities: commentary

**DOI:** 10.1186/s42494-025-00205-7

**Published:** 2025-02-19

**Authors:** Madison Milne-Ives, Rosiered Brownson-Smith, Ananya Ananthakrishnan, Yihan Wang, Cen Cong, Gavin P. Winston, Edward Meinert

**Affiliations:** 1 Translational and Clinical Research Institute, Newcastle University, Newcastle-Upon-Tyne, NE1 7RU UK; 2 Centre for Health Technology, School of Nursing and Midwifery, University of Plymouth, Plymouth, PL4 8AA UK; 3 Department of Sport, Exercise and Rehabilitation, Faculty of Health and Life Sciences, Northumbria University, Newcastle-Upon-Tyne, NE7 7XA UK; 4 Department of Biology, University of Western Ontario, London, N6A 5B7 Canada; 5 Department of Medicine (Division of Neurology), Queen’s University, Kingston, K7L 3N6 Canada; 6 Department of Primary Care and Public Health, School of Public Health, Imperial College London, London, W12 0BZ UK

**Keywords:** Epilepsy, Intellectual disability, Artificial intelligence, Personalised treatment

## Abstract

Epilepsy is one of the most common neurological disorders, affecting more than 50 million people worldwide. Management is particularly complex in individuals with intellectual disabilities, who are at a much higher risk of having severe seizures compared to the general population. People with intellectual disabilities are regularly excluded from epilepsy research, despite having significantly higher risks of negative health outcomes and early mortality. Recent advances in artificial intelligence (AI) have shown great potential in improving the diagnosis, monitoring, and management of epilepsy. Machine learning techniques have been used in analysing electroencephalography data for efficient seizure detection and prediction, as well as individualised treatment, which facilitates timely and customised intervention for individuals with epilepsy. Research and implementation of AI-based solutions for people with intellectual disabilities and epilepsy still remains limited due to a lack of accessible long-term clinical data for model training, difficulties in communicating with people with intellectual disabilities, and ethical challenges in ensuring the safety of the AI systems for this population. This paper presents an overview of recent AI applications in epilepsy and for people with intellectual disabilities, highlighting key challenges and the necessity of including people with intellectual disabilities in research on AI and epilepsy, and potential strategies to promote the development and use of AI applications for this vulnerable population. Given the prevalence and consequences associated with epilepsy in people with intellectual disabilities, the application of AI in epilepsy care has the potential to have a significant positive impact. To achieve this impact and to avoid increasing existing health inequity, there is an urgent need for greater inclusion of people with intellectual disabilities in research around the application of AI to epilepsy care and management.

## Background

Epilepsy can increase risk of mental and physical health consequences, negatively impact quality of life, and increase risk of premature mortality [1–5]. People with intellectual disability (PwID) are significantly more likely to have epilepsy than the general population, with prevalence rates of approximately 22.5% compared to 0.5–1%, and to experience worse consequences [6–9]. Despite having significantly higher rates of treatment-resistant epilepsy (70% compared to 30%) and negative outcomes such as misdiagnosis, preventable emergency department admissions, and early mortality including sudden unexpected death in epilepsy (SUDEP) [8, 10–12], PwID are under-supported in epilepsy research and care [13]. PwID can experience significant barriers to accessing healthcare, including communication difficulties, impaired cognition, a lack of understanding from healthcare providers, and societal stigma [14, 15]. In research and care, a key issue is that PwID cannot always communicate their experiences. This is a barrier because observer reports of seizures are often unreliable, which can lead to misinterpretation of events [16] and increases the need for research into alternative methods for diagnosing and managing epilepsy for PwID. The challenge is that, as a vulnerable population who may lack capacity to consent, PwID are often excluded from clinical studies [13, 17]. Substantial under-representation of PwID and epilepsy in research has been recognised; only 5% of the publications relating to epilepsy focus on PwID and only 1.4% of presentations at “major ID conferences” related to epilepsy [13]. We conducted a review that aimed to examine remote electroencephalography (EEG) monitoring for PwID [18], but only three of the 23 included studies referred to PwID [14, 19, 20]. As novel methods for diagnosing and managing epilepsy continue to be explored, they must be designed to meet the unique needs of PwID and their impact on this population evaluated to avoid increasing the existing substantial health inequity [21].

## Main Text

### AI in epilepsy

Epilepsy is a complex condition that is poorly understood. It often presents heterogeneously, seizure occurrence is unpredictable, and a significant minority of people do not respond to anti-seizure medication [22]. Artificial intelligence (AI) techniques have been applied to address various clinical needs in epilepsy, including diagnosis, seizure detection and prediction, and management [22–26]. A strength of AI is its ability to handle complex data through analysis of large amounts of data, pattern recognition, and modelling [22, 27, 28]. Deep learning and machine learning (ML) algorithms have been developed that can analyse EEG data and recognize patterns that precede seizures, potentially allowing for real-time warnings and giving patients a greater sense of control [29–34]. They have demonstrated accuracy up to 99.6% of prediction one hour before onset [26, 35]. Accuracy can be increased through combining video, EEG, and mobile data [36] and different neural networks architectures in ML [37]. AI techniques have also been applied to improve seizure detection using wearables (with one study demonstrating “83.9% sensitivity and 35.3% false positive rate” [38]). ML techniques have also been used to integrate data from various imaging techniques to better understand how epilepsy develops [39] and to simulate intervention effects, potentially replacing less accurate animal, lesional, and cell-based models [40]. AI can also support medical and surgical decision-making [22] by using neural networks to estimate patients’ prognoses [41], classification algorithms to predict individualised response to medication [22], and deep learning algorithms to identify candidates for surgery and predict outcomes [23].

While AI has the potential to support epilepsy diagnosis and management, several challenges remain. First, due to the heterogeneous nature of seizures and limited understanding of why seizures propagate, AI models must be trained on vast amounts of data, requiring significant computational resources [22] and accessibility of high-quality, long-term datasets [25]. The lack of open-access data makes reproducibility (essential, given the risks of wrong predictions) difficult [25, 26, 42]. Other challenges include a lack of generalisability [43], developing cost-effective hardware for real-time epilepsy prediction [26], and low trust in “black box” ML [25], although this could be mitigated through the use of explainable AI [44].

### AI in epilepsy for PwID

Given the highly disproportionate prevalence of epilepsy in PwID [6–9], AI could have a profound impact, but there is limited research [14] and existing datasets are likely to be biased against this population. One study, identified by our previous review [18], compiled an EEG dataset of PwID and epilepsy and used it to develop and test a seizure detection model [14]. The study found a wide range in model performance across individuals, influenced by key factors including EEG discharge patterns, backgrounds and seizure visibility [14]. Challenges with designing a generic seizure detector for PwID included imbalanced and heterogeneous data and difficulties with annotation [14]. Another study designed a video-based AI system to record possible nocturnal seizures; it was acceptable to PwID and carers and facilitated care planning, but the study lacked a “gold standard” comparator to confirm its seizure detection accuracy [45]. While these studies represent a step forward in a highly underrepresented population, substantial effort will be required to effectively leverage AI to improve model accuracy, efficiency, and generalisability.

### Challenges with including PwID in AI and epilepsy research

Research is crucial for improving clinical outcomes [46], but the inclusion of PwID in medical research can raise ethical and practical concerns [47, 48]. Obtaining informed consent from PwID is complicated due to communication barriers and varying levels of comprehension [47, 49] and researchers may lack the time or training needed to address barriers [48]. The risk of harm is a major concern, as PwID who have communication challenges may be unable to report adverse events [50, 51]. Additional practical challenges include PwID's potential reliance on others for transport to appointments and a potential lack of exposure to research studies if their engagement with healthcare services is limited. The level of safety evidence required to justify risk–benefit decisions means that research is slower in populations that desperately need new interventions to improve clinical outcomes [47]. High prevalence of comorbidities among PwID further complicates their inclusion in epilepsy research. Comorbid conditions, such as behavioural disorders or other neurological impairments, can increase the difficulty of isolating effects [52]. This complexity often leads to challenges in evaluating intervention efficacy and safety, as outcomes may be confounded by these additional conditions [53].

### Need to include PwID in AI and epilepsy research

It is essential that these challenges be addressed. If research into the application of AI in epilepsy follows current patterns of exclusion of PwID, there is a serious risk of increasing health inequity. The disparity between new technologies and methods that have evidence for safety in the general population compared to PwID will continue to grow, and PwID—who experience higher rates of epilepsy and worse outcomes—will not benefit from improvements in care. Such potential improvements include the possibility of conducting long-term outpatient EEG recording, analysed via AI models [54, 55]. Short duration EEG options have relatively low sensitivity in epilepsy (25–56% [56], likely lower for PwID [14, 15]); long-term remote EEG or wearable monitoring could help address these issues, but produces large amounts of data [57]. These modes of seizure monitoring may be revolutionary for PwID, reducing the need for communication at the point of seizure, preventing misinterpretation, and alerting PwID and carers to take protective measures before a seizure occurs.

### Strategies to include PwID in AI and epilepsy research

There is a clear need to include PwID in epilepsy research. For research with increased patient risk, it may be appropriate to follow a step-wise approach, starting with those who have the capacity to understand and consent to participation. For research with fewer risks (e.g. wearables), it is important to consider including PwID from the outset. It is critical that, as the evidence base strengthens, research should include individuals with more profound ID, who may benefit most from advances [11]. Rather than excluding PwID, protocols should address the risks involved and establish rigorous strategies for mitigating potential harms. For example:

- ensuring a family member or primary carer is fully engaged with the study;
- including more frequent check-ins by the research team;
- adapting consent processes to support PwID in provided informed consent where possible (e.g. with support from a speech and language therapist, using a variety of materials such as Easy Read documents, videos, demonstrations, etc.);
- employing assistive communication technologies or other tailored communication methods to support PwID involvement in research studies and to detect and manage adverse events promptly; and
- providing clear, accessible information to legal-decision makers consenting on a PwID’s behalf, so that they can make a risk–benefit judgement based on their knowledge of the patient when the patient cannot be supported to provide their own consent.

Another key strategy is engaging in co-production work to identify potential issues and develop research strategies tailored to the specific needs of the target PwID population, particularly regarding new technologies like AI. Co-production involves collaborating and sharing power with key stakeholders (e.g. PwID, family and carers, healthcare professionals) to design and deliver research projects [58]and would ensure that research methodologies are designed with the needs and preferences of PwID in mind. This in turn would enhance the relevance and ethical integrity of the research. Future research could also explore how AI tools could be used to support PwID’s participation in clinical trials; for instance, by providing a personalised learning program to explain the study at the most appropriate level for the PwID or by facilitating their ability to communicate with researchers [59].

## Conclusions

AI technologies have potential to improve epilepsy diagnosis and management, but it is essential that these technologies are designed to include PwID and evaluated with PwID to avoid perpetuating existing health inequities. PwID experience higher rates of, and worse outcomes from, epilepsy, but are consistently excluded for research. As AI is increasingly incorporated into epilepsy care, we risk further widening the divide in access. Rigorous research design is needed to address concerns around consent, communication, and potential safety risks. For AI-based approaches in studies with PwID, patient and public involvement and engagement holds a great potential for tailoring the approaches to their needs and benefits, ultimately improving health outcomes in this vulnerable group.

## Data Availability

Not applicable.

## Abbreviations

AI: Artificial intelligence
EEG: Electroencephalography
ML: Machine learning
PwID: People with intellectual disability
SUDEP: Sudden unexpected death in epilepsy
